# Synergistic effects of methyl jasmonate treatment and propagation method on Norway spruce resistance against a bark-feeding insect

**DOI:** 10.3389/fpls.2023.1165156

**Published:** 2023-06-06

**Authors:** Kristina Berggren, Michelle Nordkvist, Christer Björkman, Helena Bylund, Maartje J. Klapwijk, Adriana Puentes

**Affiliations:** Department of Ecology, Swedish University of Agricultural Sciences (SLU), Uppsala, Sweden

**Keywords:** emblings, forestry, *Hylobius abietis*, *Picea abies*, plant protection, regeneration pest, somatic embryogenesis (SE)

## Abstract

Utilizing plants with enhanced resistance traits is gaining interest in plant protection. Two strategies are especially promising for increasing resistance against a forest insect pest, the pine weevil (*Hylobius abietis*): exogenous application of the plant defense hormone methyl jasmonate (MeJA), and production of plants through the clonal propagation method somatic embryogenesis (SE). Here, we quantified and compared the separate and combined effects of SE and MeJA on Norway spruce resistance to pine weevil damage. Plants produced via SE (emblings) and nursery seedlings (containerized and bare-root), were treated (or not) with MeJA and exposed to pine weevils in the field (followed for 3 years) and in the lab (with a non-choice experiment). Firstly, we found that SE and MeJA independently decreased pine weevil damage to Norway spruce plants in the field by 32-33% and 53-59%, respectively, compared to untreated containerized and bare-root seedlings. Secondly, SE and MeJA together reduced damage to an even greater extent, with treated emblings receiving 86-87% less damage when compared to either untreated containerized or bare-root seedlings in the field, and by 48% in the lab. Moreover, MeJA-treated emblings experienced 98% lower mortality than untreated containerized seedlings, and this high level of survival was similar to that experienced by treated bare-root seedlings. These positive effects on survival remained for MeJA-treated emblings across the 3-year experimental period. We conclude that SE and MeJA have the potential to work synergistically to improve plants’ ability to resist damage, and can thus confer a strong plant protection advantage. The mechanisms underlying these responses merit further examination.

## 1 Introduction

Plants with enhanced resistance traits are in demand within plant protection against pests, given the need to replace adverse methods, such as chemical pesticides, with sustainable long-term strategies (Stenberg et al., 2015; Mitchell et al., 2016; Dreischhoff et al., 2020; Lalík et al., 2020; Hernández-Suárez and Beitia, 2021). Resistance is a vital part of plant defense, as it describes a plant’s ability to avoid an attack or reduce the amount of damage received (Núñez-Farfán et al., 2007). It was recently discovered that a method used for plant propagation can make plants intrinsically more resistant to insect damage. In a study on 4-year-old Norway spruce (*Picea abies*), plants produced through somatic embryogenesis (SE) were more resistant to bark-feeding damage by the pine weevil (*Hylobius abietis*) than zygotic seedlings from the same Norway spruce families (Puentes et al., 2018). The authors found that plants propagated via SE were less frequently attacked, and received about 30% less damage by pine weevils than regular seedlings (Puentes et al., 2018). SE is a vegetative propagation method in which somatic cells or tissue is used to produce plants *in vitro* with the use of plant hormones (Mo et al., 1995; Klimaszewska et al., 2016; Egertsdotter, 2019). SE has been used for decades as a propagation method for many economically important crops (e.g., wine grapes, cacao trees, bananas) (Duarte-Aké and De-la-Peña, 2016; Etienne et al., 2016; López et al., 2022) and tree species (e.g., spruce, larch) (Lelu-Walter et al., 2013). Yet, its potential to produce conifer (and other) plants that are intrinsically more resistant to pests, has not been explored.

Given the different factors involved in producing SE plants, it is likely that the process itself affects plant resistance. For instance, initiation of the cell multiplication process and subsequent maturation of embryos requires high amounts of plant growth regulators (PGRs) such as ethylene and abscisic acid (von Aderkas et al., 2015; Méndez-Hernández et al., 2019). These plant hormones are also involved in responses to biotic stress (Müller, 2021). In some cases, somatic embryos may even be exposed to extreme pH and heat shock and, thus, often experience high levels of stress during development (Winkelmann, 2016; Méndez-Hernández et al., 2019). Such a stress stimulus early in life can prime or prepare plants for subsequent attacks, and result in faster or stronger activation of defenses (Conrath et al., 2006; Wilkinson et al., 2019). Moreover, studies have reported that plants produced via SE exhibit greater levels of secondary metabolites (which can be important for plant defense) when compared to plants produced through seeds or growing in the wild (Lamhamedi et al., 2000; Fulzele and Satdive, 2003; Domínguez et al., 2010). Producing plants via SE may, therefore, provide new opportunities to take advantage of plants’ responses to stress and reduce pest damage.

Development of strategies to enhance plant resistance against pests have focused to a great extent on the use of chemical elicitors (e.g., Walters et al., 2014; Bruce et al., 2017; Siah et al., 2018; Yassin et al., 2021). One such elicitor is the plant hormone methyl jasmonate (MeJA). MeJA is an important signaling molecule mediating stress responses in plants, and it can activate resistance mechanisms (Yu et al., 2019). Exogenous application of MeJA prior to pest exposure has been shown to reduce feeding by insect herbivores, and can result in less plant damage for example in soybean, rice, strawberry and Andean lupin (Chen et al., 2018; Senthil-Nathan, 2019; Erazo-Garcia et al., 2021; Mouden et al., 2021). Moreover, it has been shown to enhance conifer resistance against insect pests such as the pine weevil (*H. abietis*) (e.g. Puentes et al., 2021), spruce bark beetle (*Ips typographus*) (Mageroy et al., 2020a) and Japanese pine sawyer (*Monochamus alternatus*) (Chen R. et al., 2020). Treatment of conifers with MeJA has been shown to result in e.g., traumatic resin duct production and increases in terpenes and phenolic-based compounds (e.g., Krokene et al., 2008; López-Villamor et al., 2021), which are important mechanisms of tree defense. Similarly to propagation through SE, treatment with MeJA also has potential to improve forest protection against detrimental pests.

Interest in using SE as a propagation method for conifer trees and induced resistance as a forest protection method is likely to increase. In Nordic European countries, production of conifers via SE is expanding (e.g., Lelu-Walter et al., 2013; Egertsdotter et al., 2019; Rosvall et al., 2019a; Rosvall et al., 2019b), as well as the potential to use MeJA in nursery seedling production (e.g., Chen Y. et al., 2020; Nybakken et al., 2021). Given the plant protection benefits that have been documented for SE and MeJA independently, it is timely to examine the combined effects of these two factors on plant resistance. If SE plants are primed or induced during production, a second stress stimulus from MeJA could provide an even faster response and/or greater levels of resistance relative to plants that have not undergone somatic embryogenesis. Alternatively, treatment with MeJA may generate little to no response in SE relative to non-SE plants, as SE plants could already be fully primed or induced. By testing these hypotheses, it would be possible to determine if SE is compatible with other plant resistance inducing strategies such as MeJA treatment.

In this study, we experimentally compared the effects of MeJA treatment on resistance of young Norway spruce plants produced via SE or from seeds. We examined resistance to the pine weevil (*H. abietis*) since exogenous application of MeJA to Norway spruce seedlings, and other conifers, has been shown to effectively reduce damage inflicted by this insect pest (e.g., Heijari et al., 2005; Zas et al., 2014; Fedderwitz et al., 2016; Lundborg et al., 2016; Puentes et al., 2021). Furthermore, in the study by Puentes et al. (2018), which documented the plant protection benefits of SE, damage inflicted by pine weevils was used as a measure of resistance. Therefore, the pine weevil-Norway spruce system provides a suitable starting point to examine the effects of SE and MeJA together. In this study, we addressed the following questions:

1. Do SE and MeJA together increase Norway spruce resistance to pine weevil damage to a greater extent than when these two methods are used separately (i.e., are effects on resistance synergistic)?
2. What are the separate and combined effects of SE and MeJA treatment on Norway spruce survival across years in the field?

We established a field and lab experiment in which MeJA-treated and non-treated Norway spruce plants (produced via SE and from seed in nurseries) were exposed to pine weevils. Plants were followed in the lab under one growing season, and in the field for three growing seasons. We quantified the proportion of plants attacked and stem area debarked by weevils, as well as plant mortality. The field experiment allows evaluation of resistance under actual forest regeneration conditions. The lab study allows evaluation of effects under controlled and non-choice conditions, which provides insight into whether the insect is avoiding the plant or it is simply not palatable.

## 2 Materials and methods

### 2.1 Study system

The pine weevil (*Hylobius abietis* L.) is a major forest regeneration pest in Europe (Nilsson et al., 2010). They lay their eggs nearby or inside the root bark of newly-dead or dying conifers, and are thus, attracted to the odors emitted by the stumps of freshly-felled trees (Nordlander, 1991; Nordlander et al., 1997). Once forest regeneration occurs through planting, adult weevils can feed extensively on the stem bark of several conifer seedlings (Wallertz et al., 2014), often removing an entire ring of bark phloem from the stem circumference (i.e., they girdle plants). Girdling often results in seedling mortality and, consequently, large economic losses (Långström and Day, 2004; Lalík et al., 2020). Feeding takes place during the plants’ growing season (from spring till autumn in Nordic countries). Pine weevils are present in clear-cuts for up to three years after the forest is harvested, as new generations hatch after 1-2 years depending on geographical location (Bejer-Petersen et al., 1962; Nordenhem, 1989; Inward et al., 2012; Wainhouse et al., 2014), thus, feeding can occur on the same seedlings for more than one season. The parental generation stays at the clear-cut for the remaining part of their lives, but the new generation eventually leaves in search of oviposition sites (Nordenhem, 1989). Replanting due to loss of seedlings may be needed in sites with high pine weevil pressure, hence, causing increased regeneration costs (Leather et al., 1999; Mattsson, 2016).

### 2.2 Plant material

Plant material consisted of Norway spruce (*Picea abies* (L.) H. Karst) obtained from the Forestry Research Institute of Sweden (Skogforsk) and from commercial plant nurseries. Plants from Skogforsk were produced through SE (emblings hereafter), from trees belonging to the clonal archive used in breeding trials of Norway spruce. Plants were propagated via SE following the same methods as described in Puentes et al. (2018). A total of 652 emblings (~1 year old) originating from 19 full-sib families were produced, with varying number of clones per family. Zygotic seedlings (seedlings hereafter) were obtained from two commercial nurseries (Stora Enso Plantor AB in Nässja, and Södra Skogsplantor in Falkenberg, Sweden), and included seedlings of two types: smaller containerized seedlings (grown with roots in a soil plug) (n = 528, 1.5 years old) and larger bare-root seedlings (grown in an outdoor nursery bed with the opportunity to develop a larger root system) (n = 124, 3 years old). In Nordic countries, these are the two seedling types that are commercially available to forest owners for re-planting after harvest. SE plants were delivered frozen, as they were in winter storage, from Skogforsk to the University of Agricultural Sciences, Uppsala, Sweden, in May 2019. Plants were thawed by slowly increasing the temperature and then kept in a greenhouse (16h/8h light/dark and ~18/15°C day/night) until the start of the experiment. Containerized and bare-root seedlings, also previously frozen during winter storage, had already been thawed when they were received from the commercial nurseries a few days later, and placed in the same greenhouse as the emblings. Plants for the laboratory experiment were planted in 2L plastic pots, while plants for the field experiment were kept in plug trays (ø 6.5 cm per plug). After 3.5 weeks in the greenhouse, those plants intended for the field experiment were planted in the field and the remaining plants were kept in the greenhouse until laboratory trials started.

### 2.3 Experimental set-up

#### 2.3.1 Methyl jasmonate treatment

For each plant type, half of the total number of plants were treated with 10 mM methyl jasmonate (MeJA). This concentration of MeJA has been used in our previous studies (Chen et al., 2021), and shown to effectively increase resistance against the pine weevil in conifer seedlings of similar sizes (height/diameter) as those in the present study. First, MeJA (95%, Sigma-Aldrich, ref. 392707) was dissolved in ethanol; deionized water was then added to this mixture to achieve a final ethanol concentration of 2.5% (v:v). This solution was shaken vigorously until a uniform milky emulsion was obtained, and then transferred to a plastic hand-sprayer bottle (Free-Syringe PC 1.5 liter, Jape Products AB, Hässleholm, Sweden). The bottle was pumped until it reached its inner air pressure limit (2.5 bar), and shaken again before each spraying occasion. Plants were sprayed outdoors, with plants placed beside each other in two rows. The spraying nozzle was at a distance of about 30 cm from the plants, and the bottle was moved manually along each row of plants. Each plant was sprayed for about one second, with all aboveground parts being covered with the solution. Non-treated plants were similarly sprayed but with deionized water. MeJA-treated plants were kept in a separate greenhouse to avoid contamination of non-treated plants. MeJA treatment was applied on the plants designated for the field study eight or nine days prior to being planted in the field, and ten or eleven days prior to the start of each round of the lab experiment.

#### 2.3.2 Field experiment

The experimental site was located on a non-scarified clear-cut (7 ha, harvested autumn 2018, dominated by Scots pine (*Pinus sylvestris*)) near Tierp in central Sweden (60°21’N, 17°26’E) (see **Figure S4** for details). A total of 328 emblings, 228 containerized and 100 bare-root seedlings were planted in the field on 18-19 June 2019. The number of plants from each type were represented equally in both MeJA treatments (0 mM and 10 mM MeJA), with each treatment including 164 SE, 114 containerized and 50 bare-root seedlings. Stem height and basal diameter of each plant was measured the day before transferring them to the field. Average height ± standard error (and ranges) were for emblings: 31.1 ± 0.4 cm (17.0 to 48.0 cm), containerized seedlings: 29.6 ± 0.3 cm (19.5 to 38.5 cm), and bare-root seedlings: 57.0 ± 0.7 cm (40.0 to 71.0 cm). Plants were planted in nine blocks (size 7 × 8 m) with 72 plants in each block (except one larger block with 80 plants, 7 × 9 m) spread over an area of the clear-cut spanning about 90 × 80 m. Each block consisted of nine columns, and each column contained eight positions; except the larger block that consisted of ten columns and eight positions. In each block, plants were placed with a one meter distance, and with a rolling positioning of the four MeJA-treatment and plant type combinations in columns (see **Figure S1**). Plants were assigned positions in blocks based on the following four treatments. 1: MeJA-treated embling; 2: non-treated embling; 3: MeJA-treated containerized or bare-root seedling, 4: non-treated containerized or bare-root seedling, with every treatment represented twice in each column (see **Figure S1** for details). The design ensured that no plants belonging to the same treatment occurred beside each other in either a horizontal or vertical position. We also included a reference block (72 plants) with only non-MeJA-treated containerized seedlings, which allowed us to get an estimate of pine weevil pressure in the clear-cut without treatment interference. This reference block was located in close proximity to the experimental blocks.

The field experiment was a three-year study spanning from June 2019 to September 2021 (**Figure S3** for a timeline). Plants were exposed to the natural light, temperature and relative humidity and precipitation conditions of the clear-cut throughout the whole experiment. Three variables related to plant resistance were recorded: if the plant had been attacked or not by pine weevils (0 = no, 1 = yes), pine weevil stem feeding damage (area debarked), and mortality (0 = alive, 1 = dead). Inventories took place late in the growing season each year: September 2, 2019 (11 weeks after planting; all three variables), September 15, 2020 (attack and mortality), and September 29, 2021 (attack and mortality) (see **Figure S3** for an overview of the timeline and variables recorded). To estimate total area debarked per plant we measured the following variables: (1) debarked height - the height from the ground (right above the root collar) to the upper side of the uppermost pine weevil feeding scar on the stem, and (2) percentage debarked - the proportion of stem area damaged (%) in relation to the total surface area up to the debarked height described in (1). Using these measurements and the equation for the circumference of a circle (which estimates the perimeter of the plant stem), we calculated the debarked area (cm^2^) for each plant as: Total area debarked = Circumference of the stem (π·d) × (debarked height × percentage debarked). If the percentage debarked was found to be less than 10%, stem area debarked was calculated by measuring the area of each scar using graded millimeter templates and adding up these scars (cm^2^) (see **Figures S6**, **S7** for pictures of pine weevil feeding damage).

#### 2.3.3 Laboratory experiment

A total of 324 emblings, 300 containerized and 24 bare-root seedlings were used in the laboratory experiment. The number of plants from each type were equally represented in both MeJA treatments (0 mM and 10mM MeJA), with each treatment including 162 emblings, 150 containerized and 12 bare-root seedlings. The experiment was replicated nine consecutive times (referred to as rounds), with a new set of 72 plants each round (i.e., plants were only used once; see treatment combinations per round below) during July-August 2019. Each round was three or four days long. Stem height and basal diameter of the individual plant was measured in the morning, or one day before the start of each round (see **Figure S3** for an overview of the timeline and variables recorded). Average plant height ± standard error (and ranges) were for emblings: 40.8 ± 0.4 cm (17.0 to 60.0 cm), containerized seedlings: 33.7 ± 0.3 cm (18.0 to 46.0 cm), and bare-root seedlings: 58.5 ± 1.8 cm (36.5 to 73.0 cm).

In this non-choice test, plants were exposed to pine weevils that were collected during spring migration on May 21, 2019, at a sawmill (Balungstrands Sågverk AB) in Enviken, Sweden. Weevils were kept in a dark room at 10°C with access to water as well as stem pieces and branches of young Scots pine (*P. sylvestris*) to feed on. Seven days prior to each round, pine weevils were placed in a plastic box at room temperature and natural light (~25°C, light/dark: 16h/8h), for acclimatization, with Scots pine branches and water. Three to four days before the start of a round, food was removed in order to starve the pine weevils. During a round, each plant was obligatorily exposed to one starved pine weevil for three or four days, depending on how fast they started feeding. Note that plants in the same round were exposed to the same number of days to pine weevils, but the number of exposure days differed between rounds. A plastic transparent cylinder with mesh net on the top opening (h: 64 cm, d: 14 cm), enclosed each potted plant along with a pine weevil that had access to water (see **Figure S5** for details). The experiment was conducted in a lab (Swedish University of Agricultural Sciences, Uppsala, Sweden) under room temperature conditions (~25 °C) with natural light coming in from the large windows of the lab (no artificial lamps were used). Plants were placed closely together in rows on tables, and the same within-block rolling treatment order as in the field was used (treatment 1: MeJA-treated embling; 2: non-treated embling; 3: MeJA-treated containerized or bare-root seedling, 4: non-treated containerized or bare-root seedling). Every round had a different order of treatments in columns/positions from the previous one. After each round ended, cylinders and pine weevils were removed, and the stem of each plant was cut right below where the lowest feeding scar was found on the stem (most often close to the root collar). Stems were kept in a refrigerator (5 °C) until damage was scored (maximum within 7 days), and then discarded.

We recorded whether the plant had been attacked or not by the pine weevil, as well as pine weevil feeding damage to the stem (area debarked) (see **Figures S6**, **S7** for pictures of pine weevil feeding damage). The debarked area was calculated for each plant by measuring each feeding scar using graded millimeter templates, and adding all areas together (cm^2^). Each plant in the laboratory experiment was only scored once.

### 2.4 Statistical analyses

All analyses were conducted in R version 4.2.2. (R Core Team 2022). Linear mixed models were fitted with the *lmer*-function and generalized linear mixed models with the *glmer*-function from the *lme4* package (Bates et al., 2015). Models were validated by inspecting residuals *vs*. predicted values, and using Levene’s test for examining equal variances across treatments (*LeveneTest*-function; *car* package (Fox & Weisberg, 2019)) and by simulating and plotting scaled residuals using the *DHARMa* package (Hartig, 2021). Significance of main effects and interactions was tested with analysis of deviance using the *Anova* command from the *car* package (Fox and Weisberg, 2019). Estimated means for each treatment level and combinations were obtained through *emmeans* in the *emmeans* package (Lenth et al., 2020). Multiple comparisons were conducted between treatment means using the Tukey adjustment in the *emmeans* package.

#### 2.4.1 Field experiment

To examine the effects of plant type and MeJA treatment on the proportion of plants attacked (0 = no, 1 = yes) by pine weevils and plant mortality (0 = alive, 1 = dead) by the end of the first year (September 2019), we fitted generalized linear mixed models with a binomial distribution. Similarly, to examine the effect of plant type and MeJA on area debarked we fitted a linear mixed model. Plants that had received zero damage were excluded from the model, and area debarked by pine weevils (cm^2^) was log-transformed to meet model assumptions. For all these models, plant type (containerized seedling, bare-root seedling and embling), MeJA treatment (0 mM and 10 mM) and their interaction, were used as fixed effects. Initial plant height (height at the start of the experiment) was also included as a continuous covariate, and block was included as a random effect. The effects of treatment on the 19-full sib SE-families of Norway spruce used in the experiments were not examined separately, as these families responded similarly to MeJA treatment in the field (**Figure S2**).

Effects of plant type and MeJA on non-cumulative mortality in September 2020 (referred to as year 2), and non-cumulative and cumulative mortality in September 2021 (referred to as year 3) were analysed using generalized linear mixed models with a binomial distribution. These models included the same fixed and random effects as described above for attack, area debarked, and mortality. In analyses of non-cumulative mortality, plants that had died the previous year were excluded. Thus, these models examined mortality that occurred only that year (2020 or 2021). Moreover, since all containerized seedlings had practically died by the second year (97% mortality, 5 plants alive), these were excluded in the analyses of non-cumulative mortality for years 2020 and 2021 (i.e., plant type included only emblings and bare-root seedlings). On the other hand, analyses of cumulative mortality in 2021 represented the total plant mortality for the duration of the whole experiment (across 3 years) for all treatment combinations (i.e., no plant types were excluded).

#### 2.4.2 Lab experiment

The effects of plant type and MeJA on proportion attacked were analysed using a generalized linear mixed model with a binomial distribution. Area debarked by pine weevils (cm^2^) was log-transformed and effects were analysed with a linear mixed model. For both models, plant type (containerized seedling, bare-root seedling and embling), MeJA treatment (0 mM and 10 mM) and their interaction, were used as fixed effects. Plant height (height at the start of the round) was also included as a continuous covariate, and round (replication in time) was included as a random effect.

#### 2.4.3 Calculations of additive, synergistic or antagonistic effects of SE and MeJA

To determine the magnitude and direction of the effect on plant resistance when MeJA and SE occur together, we calculated if the effect was additive, synergistic or antagonistic. An interaction is additive when their combined effect is the sum of each independent effect, and it is synergistic or antagonistic when their combined effect is greater or smaller (respectively) than the sum of each independent effect. Observed effects of seedlings and emblings exposed to pine weevils (i.e., actual values of area debarked per plant), were compared to expected effects obtained from the statistical model for area debarked, following the method used in Bansal et al., 2013 (see **Supplementary Material**, section 1.3 **Supplementary Text**). Calculations were only made for pine weevil damage recorded the first year in the field, and comparisons of observed and expected effects were conducted separately using the two types of control treatment plants (untreated containerized and bare-root seedlings).

## 3 Results

### 3.1 Field experiment (year 1)

#### 3.1.1 Reference block

Overall, pine weevil pressure was high at the clear-cut where the experiment was located, as indicated by the levels of damage in the reference block. The reference block contained only non-MeJA-treated containerized seedlings of Norway spruce, and was situated close to the experimental blocks. The first year, late in the season (September 2019; 11 weeks after planting), 96% of the plants in the reference block had been attacked, resulting in 93% mortality. Stem area debarked ranged from 1.2 to 20.7 cm^2^ (average wound size per plant ± standard error: 7.8 ± 0.9 cm^2^) for plants in this block. By the second year, only two plants were alive in the reference block, and by the third year, all were dead.

#### 3.1.2 Proportion attacked

**Figure 1:**
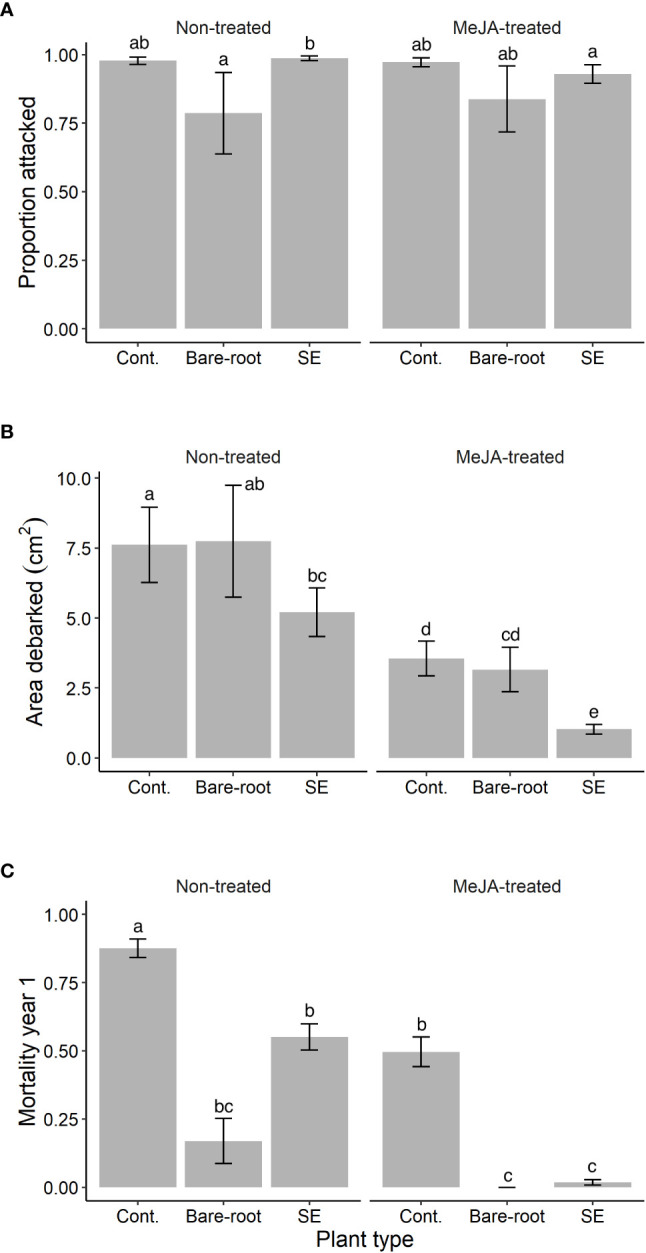
Estimated means (± standard errors) of **(A)** the proportion of Norway spruce (*Picea abies*) plants attacked, and **(B)** area debarked (cm^2^) by pine weevils (*Hylobius abietis*), as well as **(C)** plant mortality (proportion that died) by September 2019, year 1 of the field experiment. Treatments represent different plant types (Cont. = containerized seedlings, Bare-root = bare-root seedlings, SE = emblings produced via somatic embryogenesis) treated (or not) with the plant hormone methyl jasmonate (Non-treated; MeJA-treated = 10 mM sprayed once in June, 2019). Sample sizes for each treatment from left to right: Non-treated containerized seedlings n=114; bare-root seedlings n=50; emblings n=164; MeJA-treated containerized seedlings n=114; bare-root seedlings n=50; emblings n=164. Different letters indicate significantly different means. A table with pairwise comparisons and p-values can be found in the **Supplementary material (Table S1)**.

In the first year, attack was in general high with 93% of all experimental Norway spruce plants being attacked by pine weevils. We found that the proportion of plants attacked differed significantly between treatment combinations (significant plant type × MeJA interaction, **Table 1**). Among non-treated plants, emblings were similarly attacked by pine weevils when compared to containerized seedlings (**Table S1**; **Figure 1A**). Yet, they were attacked to a greater extent (25% more) than bare-root seedlings (**Table S1**; **Figure 1A**). Even though the same pattern was observed for MeJA-treated plants (**Figure 1A**), differences in attack between treated plant types were not statistically significant (**Table S1**). Nonetheless, treatment with MeJA significantly reduced attack for emblings (6% reduction) relative to non-treated emblings (**Table S1**; **Figure 1A**).

**Table 1:** Summary of results from models examining the effects of plant type and MeJA treatment on pine weevil attack and area debarked, and plant mortality, in the field experiment the first year (September 2019).

Field year 1	Attack			Area debarked			Mortality		
	χ^2^	df	p-value	χ^2^	df	p-value	χ^2^	df	p-value
Plant type	8.71	2	**0.013**	11.55	2	**0.003**	37.38	2	**< 0.00001**
MeJA treatment	0.16	1	0.692	33.98	1	**< 0.00001**	32.97	1	**< 0.00001**
Plant type × MeJA	6.87	2	**0.032**	28.13	2	**< 0.0001**	10.43	2	**0.005**
Plant height	4.82	1	**0.028**	5.95	1	**0.015**	0.04	1	0.850

χ^2^: Chi-square value; df: degrees of freedom; p-value; plant type (containerized seedlings, bare-root seedlings and emblings of Norway spruce); MeJA (methyl jasmonate) treatment (0 mM and 10 mM); attack (0 = no, 1 = yes); area debarked (cm^2^); plant mortality (0 = alive, 1 = dead). Plant height (at the time of planting) was included as a covariate, and blocks in the field were included as a random effect (not shown). Significant effects (p < 0.05) are in bold.

#### 3.1.3 Area debarked

We found that area debarked by pine weevils was affected by plant type and MeJA treatment, both separately and in combination (**Table 1**). Among untreated plants, emblings received the lowest levels of damage, 32% and 33% less than containerized and bare-root seedlings respectively (**Figure 1B**). However, these differences were statistically significant only when comparing emblings to containerized seedlings (**Table S1**). Treatment with MeJA reduced damage for all plant types, but damage reduction was much greater for emblings than for any other plant type (**Figure 1B**). Emblings, containerized and bare-root seedlings experienced an 80%, 53% and 59% reduction in damage, respectively, when each was compared to its own untreated plant group. Moreover, we found that SE and MeJA together resulted in an 86% and 87% reduction in damage, when MeJA-treated emblings were compared to non-treated containerized and bare-root seedlings, respectively (**Figure 1B**). Pairwise comparisons indicated that mean area debarked for MeJA-treated emblings was significantly lower than all other treatment means (**Table S1**). In addition to area debarked, we also noted that the average bark wound size inflicted by pine weevils for MeJA-treated emblings was much smaller than that of non-treated containerized seedlings (average wound size per plant type ± standard error, MeJA-treated emblings: 1.0 ± 0.2 cm^2^, non-treated containerized seedlings: 7.6 ± 1.3 cm^2^).

#### 3.1.4 Additive, synergistic or antagonistic effects of SE and MeJA on area debarked

We compared the observed and expected effects of SE and MeJA on area debarked. We estimated these effects using the two types of control treatment plants, untreated containerized and bare-root seedlings, separately (see **Supplementary materials**). Relative to containerized seedlings, we found that the difference between the observed and expected effect of SE and MeJA on area debarked was positive (Obs – Exp = 0.127; **Figure S8**). Furthermore, the lower 95% confidence limit of the difference was greater than zero (lower CI: 0.091; **Figure S8**). Likewise, relative to bare-root seedlings, the difference was also positive (Obs – Exp = 0.083; **Figure S8**) and the 95% confidence limit was greater than zero (lower CI: 0.047; **Figure S8**). According to Bansal et al. (2013), this indicates that the effects of SE and MeJA together on plant resistance were synergistic, i.e., much greater than the sum of the independent effects.

#### 3.1.5 Mortality

By September of the first year, late in the season, 39% of all experimental plants had died. However, mortality was significantly different among plant types, MeJA treatment and the combination of these two factors (**Table 1**). Among untreated plants, emblings experienced a significant 37% reduction in mortality relative to containerized seedlings, but died to a much greater extent (224% more) relative to bare-root seedlings (**Table S1**; **Figure 1C**). If plants were treated with MeJA, mortality was significantly reduced (**Table 1**). Relative to each untreated plant group, mortality was decreased by 97%, 43% and 100% for MeJA-treated emblings, containerized and bare root seedlings, respectively. Compared to plants receiving no treatment, SE and MeJA together significantly diminished mortality by 98% and 89% relative to untreated containerized and bare-root seedlings respectively (**Table S1**; **Figure 1C**).

### 3.2 Laboratory experiment

#### 3.2.1 Proportion attacked

**Figure 2:**
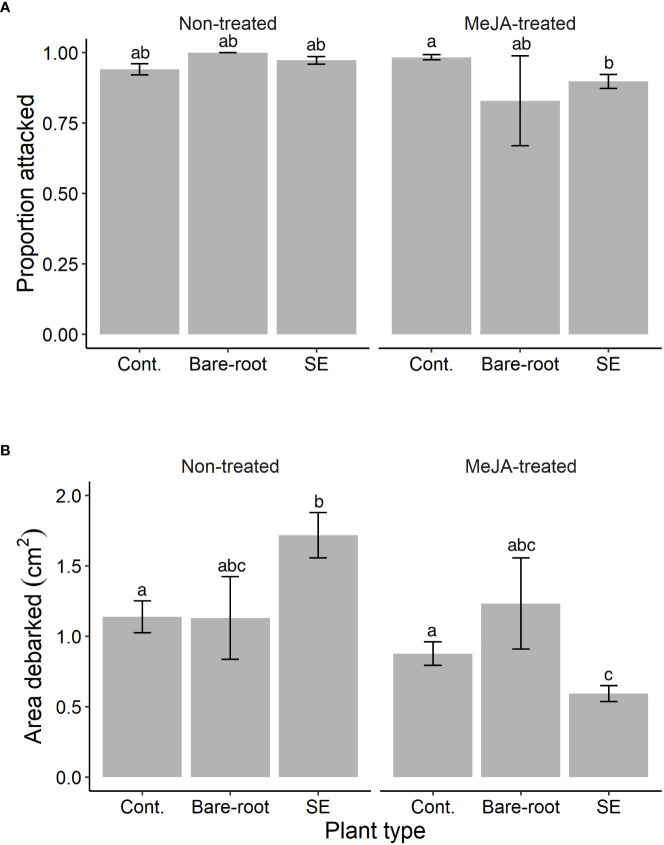
Estimated means (± standard errors) of **(A)** the proportion of Norway spruce (*Picea abies*) plants attacked and **(B)** area debarked by pine weevils (*Hylobius abietis*) in the lab experiment (replicated in time between July-August 2019). Treatments represent different plant types (Cont. = containerized seedlings, Bare-root = bare-root seedlings, SE = emblings produced via somatic embryogenesis) treated (or not) with the plant hormone methyl jasmonate (Non-treated; MeJA-treated = 10 mM sprayed once between July and August, 2019). Sample sizes for each treatment from left to right: Non-treated containerized seedlings n=150; bare-root seedlings n=12; emblings n=162; MeJA-treated containerized seedlings n=150; bare-root seedlings n=12; emblings n=162. Different letters indicate significantly different means. A table with pairwise comparisons and p-values can be found in the **Supplementary material (Table S2)**.

Similar to the field, the proportion of plants attacked by pine weevils was also high for the lab experiment, with 94% of all Norway spruce plants being attacked. We found that attack differed significantly between treatment combinations (significant plant type × MeJA interaction, **Table 2**). Among untreated plants, all plant types were similarly attacked (**Table S2**; **Figure 2A**). Among MeJA-treated plants, emblings experienced 9% less attack than containerized seedlings (**Table S2**), but were similarly attacked to bare-root seedlings. Bare-root seedlings experienced the greatest reduction in attack (17% less) compared to untreated plants of the same type (**Figure 2A**).

**Table 2:** Summary of results from models examining the effects of plant type and MeJA treatment on pine weevil attack and area debarked in the lab experiment (July-August 2019).

Lab experiment	Attack			Area debarked		
	χ^2^	df	p-value	χ^2^	df	p-value
Plant type	1.78	2	0.410	19.21	2	**< 0.001**
MeJA	4.15	1	**0.042**	7.77	1	**0.005**
Plant type × MeJA	9.93	2	**0.007**	43.21	2	**< 0.00001**
Plant height	3.63	1	0.057	26.22	1	**< 0.0001**

χ^2^: Chi-square value; df: degrees of freedom; p-value; plant type (containerized seedlings, bare-root seedlings and emblings of Norway spruce); MeJA (methyl jasmonate) treatment (0 mM and 10 mM); attack (0 = no, 1 = yes); area debarked (cm^2^). Plant height (at the time of each experimental round) was included as a covariate, and round (replication in time) was included as a random effect (not shown). Significant effects (p < 0.05) are in bold.

#### 3.2.2 Area debarked

We found that pine weevil damage differed among treatment combinations (significant plant type × MeJA interaction, **Table 2**), but the pattern of damage was somewhat different than that of the field experiment. Among untreated plants, emblings received the most damage, 51% and 52% more than containerized and bare-root seedlings respectively (**Figure 2B**). MeJA treatment significantly reduced damage levels for emblings and containerized seedlings by 66% and 23% respectively, relative to untreated plants of the same group (**Table S2**; **Figure 2B**). Damage to bare-root seedlings was slightly higher when plants of this type were MeJA-treated, but this difference was not significant (**Table S2**; **Figure 2B**). Similar to the field, SE and MeJA together resulted in the lowest plant damage levels relative to all treatments (**Table S2**). Area debarked was 48% lower for treated emblings when compared to either untreated containerized or bare-root seedlings (**Table S2**). No plants in the laboratory experiment died during the duration of each experimental round.

### 3.3 Field mortality years 2 and 3

**Figure 3:**
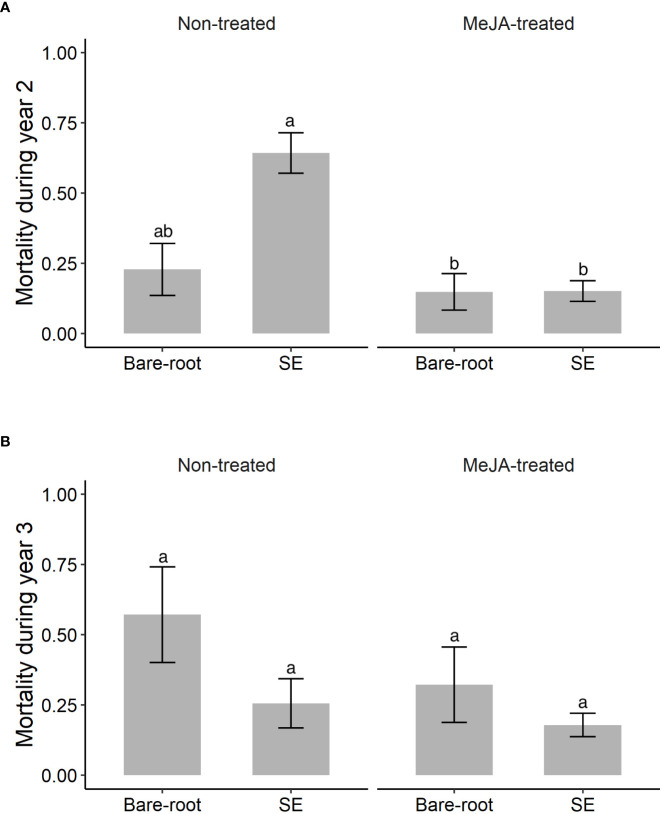
Estimated means (± standard errors) of non-cumulative mortality of Norway spruce (*Picea abies*) plants (proportion that died) by **(A)** year 2 (September 2020) and **(B)** year 3 (September 2021) of the field experiment. Due to the high mortality of containerized seedlings after year 1, this plant type was excluded from the analyses for years 2 and 3. Treatments represent different plant types (Bare-root = bare-root seedlings, SE = emblings produced via somatic embryogenesis) treated (or not) with the plant hormone methyl jasmonate (Non-treated; MeJA-treated = 10 mM sprayed once in June, 2019). Sample sizes for each treatment from left to right for years 2 and 3 respectively: Non-treated bare-root seedlings n=42, n=22; emblings n=73, n=35; MeJA-treated bare-root seedlings n=50, n=33; emblings n=161, n=143. Different letters indicate significantly different means. A table with pairwise comparisons and p-values can be found in the **Supplementary material (Table S3)**.

During the second and third year of the field experiment, attack rate of Norway spruce plants by pine weevils remained high. Among those plants that were alive during the second and third year, 93% were attacked during year 2 and 71% during year 3. In addition, late in the season during the second and third year, 33% and 26% of the previous year’s surviving plants had died. However, mortality differed among plant type and MeJA treatment combinations for year 2, but not for year 3 (**Table 3**). Note that since all containerized seedlings had practically died by the second year (97% mortality, 5 plants alive), these were excluded from analyses of non-cumulative mortality in years 2 and 3. In year 2, untreated emblings experienced 182% greater mortality than untreated bare-root seedlings (**Table S3**; **Figure 3A**). MeJA treatment significantly diminished mortality for treated emblings (77% less) relative to untreated plants of this group (**Table S3**). Together SE and MeJA resulted in 34% reduction in mortality when treated emblings were compared to untreated bare-root seedlings, resulting in these two groups having similar mortality levels (**Table S3**; **Figure 3A**). In year 3, mortality of plants that had survived the previous year was similar for plant type and MeJA treatment combinations (**Table 3**). MeJA treatment reduced damage for both emblings and bare-root seedlings by 30% and 44% respectively (**Figure 3B**), but these differences were not statistically significant (**Table S3**).

**Table 3:** Summary of results examining the effects of plant type and MeJA treatment on plant mortality during years 2 and 3 in the field (September 2020 and 2021, respectively), as well as years 1 to 3 (September 2019 to 2021).

Mortality field	Year 2 *non-cumulative*			Year 3 *non-cumulative*			Years 1-3 *cumulative*		
	χ^2^	df	p-value	χ^2^	df	p-value	χ^2^	df	p-value
Plant type	6.33	1	**0.012**	2.03	1	0.154	12.01	2	**0.002**
MeJA	1.46	1	0.228	3.05	1	0.081	8.55	1	**0.003**
Plant type × MeJA	9.89	1	**0.002**	0.58	1	0.445	7.54	2	**0.023**
Plant height	7.57	1	**0.006**	0.34	1	0.559	1.15	1	0.284

χ^2^: Chi-square value; df: degrees of freedom; p-value; plant type (containerized seedlings, bare-root seedlings and emblings of Norway spruce) and MeJA (methyl jasmonate) treatment (0 mM and 10 mM); plant mortality (0 = alive, 1 = dead). Mortality for years 2 and 3 was analyzed as non-cumulative (i.e., only plants that were alive in September the previous year were included in analyses). Due to high mortality of containerized seedlings after year 1, this plant type was not included in analyses for years 2 and 3. For years 1-3, mortality was analysed as cumulative (i.e., total mortality across the three years for all plant types). Plant height (at the time of planting) was included as a covariate, and blocks in the field were included as a random effect (not shown). Significant effects (p < 0.05) are in bold.

**Figure 4:**
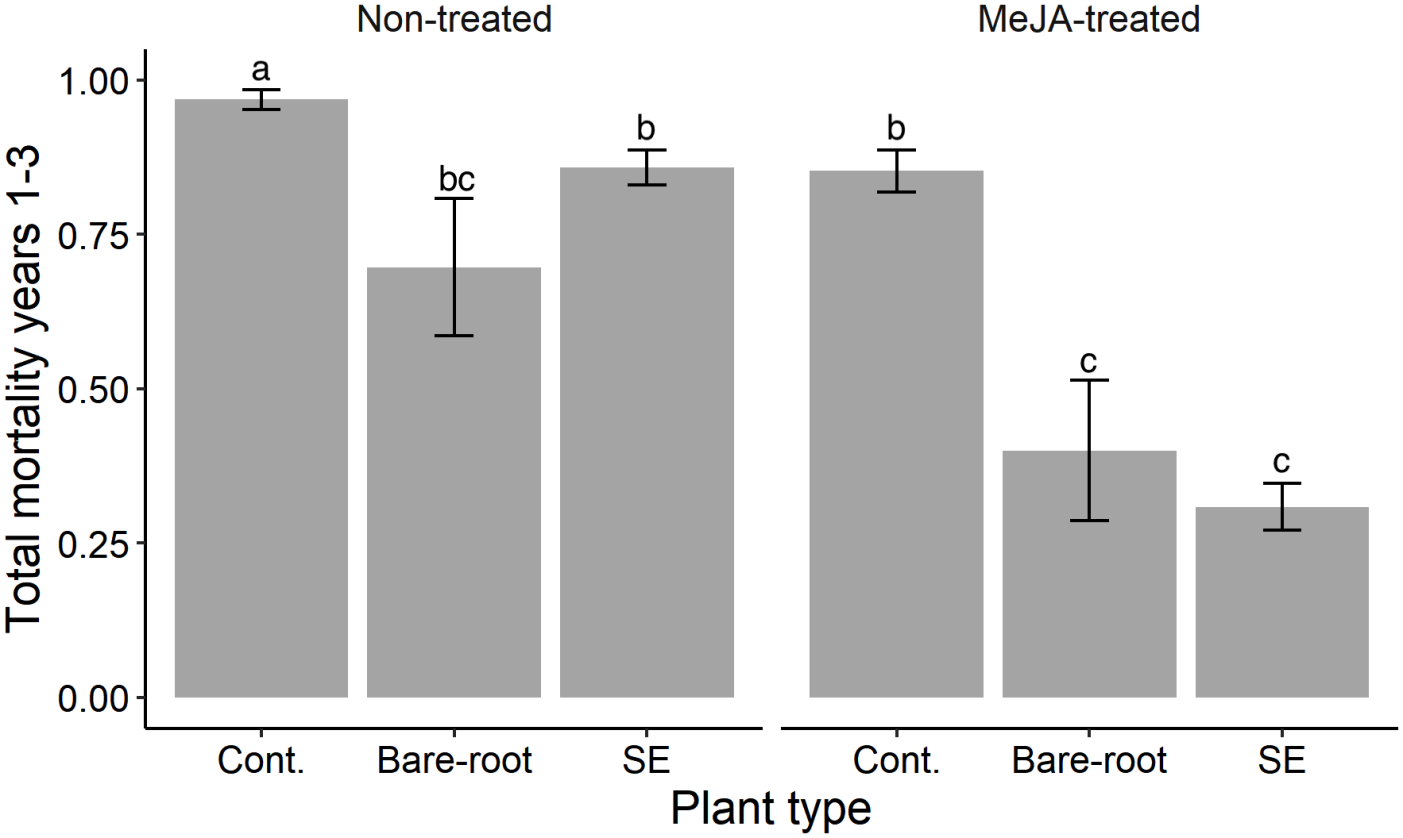
Estimated means (± standard errors) of cumulative mortality of Norway spruce (*Picea abies*) plants over the entire experimental period in the field (years 1-3). Treatments represent different plant types (Cont. = containerized seedlings, Bare-root = bare-root seedlings, SE = emblings produced via somatic embryogenesis) treated (or not) with the plant hormone methyl jasmonate (Non-treated; MeJA-treated = 10 mM sprayed once in June, 2019). Sample sizes for each treatment from left to right: Non-treated containerized seedlings n=114; bare-root seedlings n=50; emblings n=164; MeJA-treated containerized seedlings n=114; bare-root seedlings n=50; emblings n=164. Different letters indicate significantly different means. A table with pairwise comparisons and p-values can be found in the **Supplementary material (Table S3)**.

Overall, across the 3 years, 70% of all the experimental plants planted in year 1 had died. Cumulative mortality was significantly lowest for MeJA-treated emblings (**Table S3**; **Figure 4**). Of all treated emblings, 31% had died by the end of the experiment, which translated into a 68% lower mortality compared to untreated containerized seedlings. Treated bare-root seedlings experienced the second lowest mortality (40%), and the highest mortality was recorded for untreated containerized seedlings (97%) (**Figure 4**). Alone, SE significantly diminished mortality by 11% when comparing untreated emblings to untreated containerized seedlings (**Table S3**). Likewise, MeJA-treatment significantly decreased mortality by 64%, 12% and 43% for treated emblings, containerized and bare-root seedlings respectively when compared to each untreated group (**Table S3**).

## 4 Discussion

Our study found that producing plants via SE and subsequently treating them with MeJA can increase Norway spruce resistance to pine weevil damage to a greater extent than when these two occur separately. Together, SE and MeJA decreased damage by 86-87% when treated emblings were compared to either untreated containerized or bare-root seedlings in the field, and by 48% in the lab. Moreover, survival in the field was positively affected by SE and MeJA together. MeJA-treated emblings experienced 98% and 89% lower mortality during the first year relative to untreated containerized and bare-root seedlings, respectively. These positive effects on survival remained for MeJA-treated emblings across the three years that plants were followed. Overall, we conclude that SE and MeJA have the potential to work synergistically to improve plants’ ability to resist and survive damage, and can thus confer a strong plant protection advantage.

### 4.1 Effects of SE and MeJA on plant resistance and mortality in the field year 1

SE and MeJA, separately and in combination, affected to different extents the proportion of Norway spruce plants attacked by pine weevils, stem area debarked and survival across the experimental period. Among non-MeJA treated plants, emblings were attacked similarly to containerized seedlings, but attacked to a greater extent than bare-root seedlings (**Table S1**; **Figure 1A**). These results suggest that SE alone does not necessarily diminish the likelihood of plants being attacked by pine weevils. In contrast, Puentes et al. (2018) found a 10% reduction in attack for non-MeJA treated Norway spruce emblings relative to seedlings in one of their trials. It is important to consider that pine weevil pressure in the present study was very high (93% of plants were attacked the first year), which can make it harder to detect preferences among plant types (e.g., Tudoran et al., 2021). Indeed, Puentes et al. (2018) only found differences in attack between emblings and seedlings in the trial with lower pine weevil pressure (41% of plants were attacked), and no difference in the trial with almost 100% attack. On the other hand, when emblings were treated with MeJA, we found that the proportion of plants attacked decreased significantly by 6% compared to untreated emblings (**Table S1**). These positive effects were only seen for emblings, as containerized and bare-root seedlings had similar attack levels both in the untreated and treated groups (**Table S1**; **Figure 1A**). A lack of effect of MeJA treatment on the proportion of plants attacked is in line with Zas et al. (2014). The authors found that treating Norway spruce, Scots pine and Monterey pine seedlings with MeJA did not reduce the likelihood of being attacked by pine weevils in the field. Overall, it appears that SE and MeJA alone have little to no effect on plant attractiveness to pine weevils. Together, these two factors may lower the probability of being attacked, but the magnitude of these potential effects appears to be small. Evaluation of pine weevil preferences under controlled conditions (e.g., in an olfactometer), in addition to measuring volatile emissions for plants in each treatment, would be needed to disentangle the underlying causes of the observed pattern.

Even though there were small differences in the proportion of plants attacked, we found large differences in stem area debarked by pine weevils among treatment combinations (**Figure 1B**). In line with previous studies, we corroborated that MeJA treatment alone can effectively reduce pine weevil damage to conifer seedlings (Zas et al., 2014; Chen Y. et al., 2020; Puentes et al., 2021). On its own, we found that MeJA could decrease field damage the first year by about 50% on average for both types of Norway spruce nursery seedlings. Likewise, SE alone reduced damage to Norway spruce emblings by roughly 30% compared to seedlings produced by seed, as also reported by Puentes et al. (2018). Together, SE and MeJA acted synergistically to reduce stem area debarked (**Figure S8**), with treated emblings receiving 86-87% less damage than untreated containerized and bare-root seedlings. The traits and mechanisms underlying these effects need to be uncovered in subsequent studies, but a few explanations could be put forward and are discussed below.

Firstly, it seems that the lower levels of damage received by MeJA-treated emblings cannot be fully explained by a lower probability of being attacked (**Figure 1A**). Therefore, it is likely that differences in plant palatability, rather than attractiveness to pine weevils are more important. In line with this, we found that feeding wounds inflicted by pine weevils were much smaller on average (86% smaller) for MeJA-treated emblings relative to untreated containerized seedlings. Lower feeding rates may be due to enhanced chemical and/or other defenses in treated emblings, which deter pine weevils. For instance, plants produced through SE have been shown to harbor greater levels of secondary compounds than their non-SE counterparts (Lamhamedi et al., 2000; Fulzele and Satdive, 2003). Likewise, treatment with MeJA can result in traumatic resin duct production and increases in terpene and phenolic-based compounds (e.g., Martin et al., 2002; Krokene et al., 2008; López-Villamor et al., 2021; Puentes et al., 2021). Therefore, SE and MeJA may have a compounded effect on plant chemistry (and/or other traits), which exceeds the effect of each factor alone.

Greater resistance of treated emblings may occur due to a double-priming or induction of defenses; first early in life through SE (i.e., embryos are exposed to stress), and later through exogenous MeJA application. If plants have previously experienced stress, they can become more resistant to subsequent attacks through two mechanisms: 1) prolonged up-regulation of inducible defenses, and 2) priming of defenses (Wilkinson et al., 2019). In the first case, defenses are kept upregulated (i.e., active) for weeks or months following the stress stimulus. For example, newly-formed leaves of tomato plants have greater trichome densities in the weeks following MeJA treatment, relative to untreated plants (Boughton et al., 2005). However, such a strategy can be very costly for plants and is often not sustained for long periods of time. In the second case, defenses are primed and maintained at slightly induced levels, and become rapidly activated upon subsequent attack (Wilkinson et al., 2019). Since this strategy is less resource-costly, defenses can remain primed for longer periods of time. Our study does not allow us to distinguish if up-regulation and/or priming of defenses is responsible for the synergistic effect of SE and MeJA. However, it has been shown that MeJA can act as both an up-regulating and a priming agent in Norway spruce (Mageroy et al., 2020a). To conclusively determine the underlying mechanisms, a study on the effects of SE and MeJA on defense priming/induction, e.g., by examining defense gene transcription as in Mageroy et al. (2020b), would be needed.

Treating Norway spruce emblings with MeJA did not only reduce damage to a greater extent than the other treatments, but also significantly reduced plant mortality. During the first year, treated emblings experienced only 2% mortality compared to non-treated containerized seedlings, which experienced 88% mortality (**Figure 1C**). Such dramatic reduction in embling mortality was not expected, given the high pine weevil pressure at the field site (93% of plants died in the reference block), and that SE and MeJA individually decreased mortality by roughly 40% (**Figure 1C**). Mortality due to pine weevil feeding is often caused by removal of an entire ring of bark from the stem circumference (i.e., girdling). Girdling disrupts or hinders nutrient transport through the phloem (Romero, 2014), which can lead to plant death. Treatment with MeJA alone has been shown to reduce the likelihood of girdling by pine weevils, and therefore, increase conifer seedling survival (Zas et al., 2014; Fedderwitz et al., 2016). More specifically, Fedderwitz et al. (2016) showed that feeding scars are more spread out across the stem in MeJA-treated relative to untreated seedlings. Pine weevils often concentrate their feeding to the basal part of the stem, but treatment with MeJA appears to make seedlings less palatable, which changes their feeding behavior (Fedderwitz et al., 2016). In line with this, we also observed (but did not measure) that treated emblings tended to have shallower feeding scars (i.e., bark wounds did not always reach the stem wood) relative to untreated seedlings (K. Berggren, pers. obs.). Hence, the positive effects of SE and MeJA together on plant survival are probably mediated by the reduction in stem area debarked, and thus, lower likelihood of girdling for these plants.

Differences in mortality among treatments could also be a result of variation in size among plant types. Bare-root plants experienced the lowest mortality rates of all plant types (**Figure 1C**), and these plants were also the largest and thickest in terms of stem height and diameter. A previous study has shown that there is a positive relationship between Norway spruce basal diameter and survival to pine weevil damage (Thorsen et al., 2001), indicating that thicker stems can confer greater tolerance to damage. These positive effects could be mediated by physical bark properties that hinder girdling in thicker stems, and/or that larger and vigorous plants are better at recovering from stem damage (e.g., Neely, 1988; Boyes et al., 2019). In our experiment, bare-root plants received similar levels of pine weevil damage as containerized seedlings, both in the untreated and MeJA-treated group (**Figure 1B**). This indicates, firstly, that the lower mortality of bare-root seedlings relative to containerized seedlings is likely due to their size and not the amount of damage received. Secondly, that the effects of SE and MeJA on plant survival (and resistance) were not mediated by size differences since emblings were much smaller than bare-root seedlings (on average 30 cm *vs*. 50 cm, respectively; see Materials and methods). Yet, SE and MeJA together lowered mortality to the same extent as if a thicker and larger plant was planted. From a practical perspective, larger plants are less convenient to handle and can be more costly to produce (Berg, 1993). Thus, a plant smaller in size and displaying similar or higher resistance as a larger plant, would be preferred from a nursery and forest regeneration perspective.

### 4.2 Effects of SE and MeJA on plant resistance in the lab

Even though we found somewhat different trends, the results from the non-choice laboratory experiment complemented those of the field. Like in the field experiment, SE alone did not seem to affect the likelihood of plants being fed upon or not by pine weevils. Untreated emblings had similar attack levels as the other plant types in the untreated group (**Figure 2A**). However, MeJA diminished attack levels for emblings, and in line with the field, these effects were small in magnitude (9% less attacked than treated containerized seedlings; **Figure 2A**). In contrast to the field, MeJA reduced attack for bare-root seedlings by 17% (**Figure 2A**), and this resulted in treated emblings and bare-root seedlings having similar attack levels on average. Overall, both lab and field experiments consistently suggest that the probability of being damaged by pine weevils is not strongly affected by SE and MeJA together.

In terms of area debarked, the pattern of damage was somewhat different than that seen in the field. Among untreated plants, emblings were most damaged by pine weevils, while bare-root seedlings received once again similar levels of damage to containerized seedlings (**Figures 1B**, **2B**). Thus, SE alone had no protective effect against damage under the lab experiment conditions. MeJA reduced damage once again for containerized seedlings and emblings but not for bare-root seedlings, which is in contrast to the field. Nonetheless, the lab and field results consistently showed that SE and MeJA together can decrease damage the most, relative to any other treatment combination (**Figures 1B**, **2B**). A few factors could help explain some of the discrepancies between the lab and field experiments. In the lab, pine weevils were previously starved and restricted to feeding on only one plant type. Adult pine weevils usually walk around in search of food; they use visual and olfactory cues, and decide to feed (or not) in close proximity (< 2.5 cm) to the plant (Nordlander, 1991; Björklund et al., 2005). The lab set-up, with plants enclosed in large plastic cylinders, may interfere with their usual feeding behavior and thus affect levels of stem area consumed. For instance, Chen et al. (2021) found that MeJA was not as effective at reducing pine weevil damage to seedlings in a non-choice 48-hour lab experiment, compared to an earlier field experiment in which MeJA significantly decreased damage (Chen Y. et al., 2020). Moreover, plants were exposed to pine weevils for a short time in the lab compared to the field experiment. Once plants are attacked, treatment effects on induced plant resistance may take more than a few days to come into play. Despite these possible interfering factors, pine weevils fed the least on treated emblings, indicating that these plants were least palatable. Therefore, both lab and field experiments provide support for the conclusion that SE and MeJA can work together to synergistically enhance Norway spruce resistance.

### 4.3 Effects of SE and MeJA on plant mortality in the field years 2 and 3

We found that SE and MeJA together significantly affected Norway spruce mortality that occurred on year 2, but not on year 3 (**Table 3**). Important to note that almost all containerized seedlings died in year 1, and we examined non-cumulative mortality only for emblings and bare-root seedlings (see Statistical analyses). Among plants that survived in year 1, mortality of untreated emblings was much greater than that of untreated bare-root seedlings in year 2 (**Figure 3A**). Thus, the positive effects of SE alone on mortality observed in year 1 no longer remained the second year. Of the few studies that have examined SE-plants across years, Grossnickle and Major (1994) found that survival of Interior spruce (*Picea glauca* (Moench) Voss × *Picea engelmannii* Parry) emblings was just as high as that of seedlings (around 90%) by the second growing season. In Puentes et al. (2018), plant mortality was not followed across years. However, we revisited the sites from Puentes et al. (2018) and found no difference in embling and seedling mortality five years after planting (K. Berggren et al., unpublished data). Our results on the effects of SE alone are in contrast to previous work, but our study does not allow us to distinguish between possible causes of plant mortality. On the other hand, the effects of SE and MeJA together on plant mortality in year 2 were in line with those found in year 1. MeJA-treated emblings continued to exhibit very low levels of mortality, similar to those of treated bare-root seedlings (**Figure 3A**). This is in line with the findings that the beneficial effects of MeJA on conifer seedlings can persist two years after treatment (Zas et al., 2014; Chen Y. et al., 2020). Among plants that survived year 2, the same pattern of lower mortality for MeJA-treated plants was observed in year 3 (**Figure 3B**), but these differences were not statistically significant (**Table 3**).

All in all, across the 3-year experimental period, the highest survival was experienced by treated emblings. Only 31% of treated emblings had died after 3 years, while 40% of treated bare-root and 97% of untreated containerized seedlings had died after this time (**Figure 4**). These results suggest that SE and MeJA together can provide beneficial effects that persist several years after treatment. Future studies should examine if these two factors not only reduce damage by pine weevils, but can also positively affect other traits important to plant survival. From a plant protection perspective, greater survival of conifer seedlings is crucial in the early years after planting when seedlings are most susceptible. Seedling vigor and survival must be high to ensure establishment of future stands. Our results corroborate that planting without any type of seedling protection can compromise successful conifer forest regeneration, as pine weevil pressure is high during the three years after harvest (Örlander and Nilsson, 1999). Our study provides a sustainable way in which to protect seedlings, and incentivizes the development of practices that take advantage of our results. For example, MeJA could be applied to emblings in nurseries, even already before plants are packaged for winter storage (e.g. Chen Y et al., 2020). Although this study focuses on Norway spruce, SE is used in the production of other conifers and plant species. Hence, examination on the effects of SE and MeJA in other species may open up plant protection possibilities beyond forestry.

## Data availability statement

The raw data supporting the conclusions of this article will be made available by the authors, without undue reservation.

## Author contributions

AP, KB, CB, HB and MK conceived and designed the experiments. KB and AP conducted the experiments and collected the data. MN conducted the statistical analyses. KB and AP wrote the manuscript with input from the co-authors. All authors contributed to the manuscript and approved the submitted version.
