# Supplementary material for: Synergistic effects of methyl jasmonate treatment and propagation method on Norway spruce resistance against a bark-feeding insect
Source: Front Plant Sci. 2023 Jun 6;14:1165156. doi: 10.3389/fpls.2023.1165156 (PMC10279954; doi:10.3389/fpls.2023.1165156)
Supplement: Supplementary file 1 [file DataSheet_1.docx]

Supplementary Material

Synergistic effects of methyl jasmonate treatment and propagation method on Norway spruce resistance against a bark-feeding insect

Kristina Berggren*, Michelle Nordkvist, Christer Björkman, Helena Bylund, Maartje Klapwijk, Adriana Puentes

*** Correspondence:** Kristina Berggren; [kristina.berggren@slu.se](mailto:kristina.berggren@slu.se)

# Supplementary Figures and Tables

## Supplementary Figures

|  | | Position | | |  | | |  | | | **Block 1** | | | |  |  | |  | |  | |
| --- | --- | --- | --- | --- | --- | --- | --- | --- | --- | --- | --- | --- | --- | --- | --- | --- | --- | --- | --- | --- | --- |
|  | **8** | | | 4A | | 1 | | 4B | 1 | | | 4A | 1 | | | 4A | 1 | | 4B | |  |
|  | **7** | | | 3B | | 2 | | 3A | 2 | | | 3B | 2 | | | 3A | 2 | | 3A | |  |
|  | **6** | | | 2 | | 3A | | 2 | 3A | | | 2 | 3A | | | 2 | 3B | | 2 | |  |
|  | **5** | | | 1 | | 4A | | 1 | 4A | | | 1 | 4B | | | 1 | 4A | | 1 | |  |
|  | **4** | | | 4B | | 1 | | 4A | 1 | | | 4A | 1 | | | 4A | 1 | | 4A | |  |
|  | **3** | | | 3A | | 2 | | 3B | 2 | | | 3A | 2 | | | 3A | 2 | | 3B | |  |
|  | **2** | | | 2 | | 3A | | 2 | 3A | | | 2 | 3B | | | 2 | 3A | | 2 | |  |
|  | **1** | | | 1 | | 4A | | 1 | 4B | | | 1 | 4A | | | 1 | 4B | | 1 | |  |
|  | | | **1** | | | **2** | **3** | | | **4** | | **5** | | **6** | | **7** | **8** | | **9** | | Column |

**Figure S1.** Example of the experimental design of blocks (or rounds) in the field and lab experiment examining differences in resistance between Norway spruce (*Picea abies*) seedlings and emblings (produced via somatic embryogenesis) that had been treated (or not) with MeJA (0 mM and 10 mM). Treatments included: 1) MeJA-treated embling, 2) non-treated embling, 3A) MeJA-treated containerized seedling, 3B) MeJA-treated bare-root seedling, 4A) non-treated containerized seedling, and 4B) non-treated bare-root seedling. Positioning of treatments per column started from the bottom left corner (column 1, position 1) with treatment 1 and upwards. When reaching the last position, the treatment that was next in turn was placed at the top of the second column (column 2, position 8) and treatment order continued downwards. Similarly, once position 1 was reached in column 2, treatments were again placed in increasing order starting from position 1 in column 3. This alternating pattern of treatments was implemented through the whole block. Note that we did not always start each block with treatment 1, but alternated between all four treatments.

**
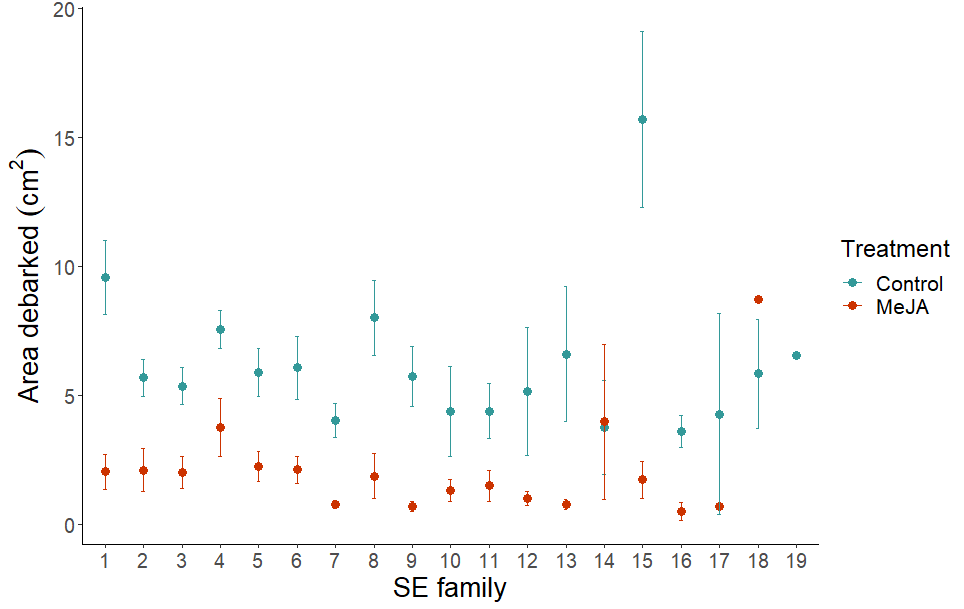
**

**Figure S2.** Arithmetic means (± standard error) of pine weevil (*Hylobius abietis*) field damage (area debarked, cm^2^) during the first year of the experiment for the 19-full sib-Norway spruce (*Picea abies*) families to which emblings originated from and were used in the experiments. Zeros were removed from the calculations of mean area debarked, so three families (17, 18, and 19) had only one individual in either the untreated or MeJA-treated group. Overall, the amounts of pine weevil damage received by the families in the field was similar when untreated (except for family 15), and became even more similar among treated emblings. With the exception of two families (14, 18), the levels of pine weevil damage decreased for all families when they were MeJA-treated relative to when they were untreated.

**
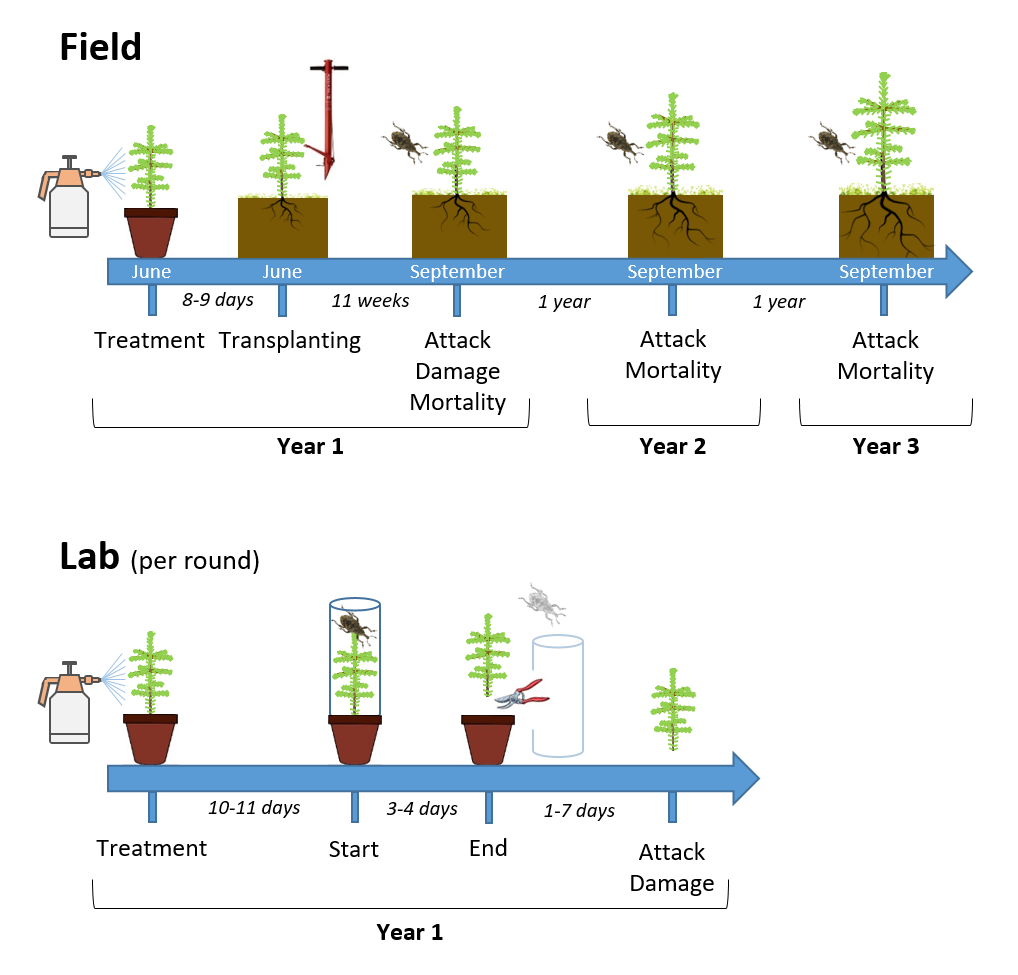
**

**Figure S3.** Overview of the experimental set-up and timeline of the field and lab experiments. The field experiment shows activities across the three years for which it was followed, while the lab shows activities for one round (9 rounds were conducted). In the field, Norway spruce (*Picea abies*) plants were treated with MeJA or with water 8-9 days prior to planting in the field. We recorded pine weevil (*Hylobius abietis*) attack, damage and plant mortality late in the season the first year, and pine weevil attack and plant mortality late in the season the second and third year. In the lab, plants were treated with MeJA or with water 10-11 days prior to start of the round. Plants were obligatorily exposed to one pine weevil for 3-4 days and were covered with a plastic cylinder to prevent the insects from escaping. At the end of the round, the cylinder and the pine weevil were removed. The aboveground part of the plants were cut and stored and 1-7 days later, pine weevil attack and damage were recorded.


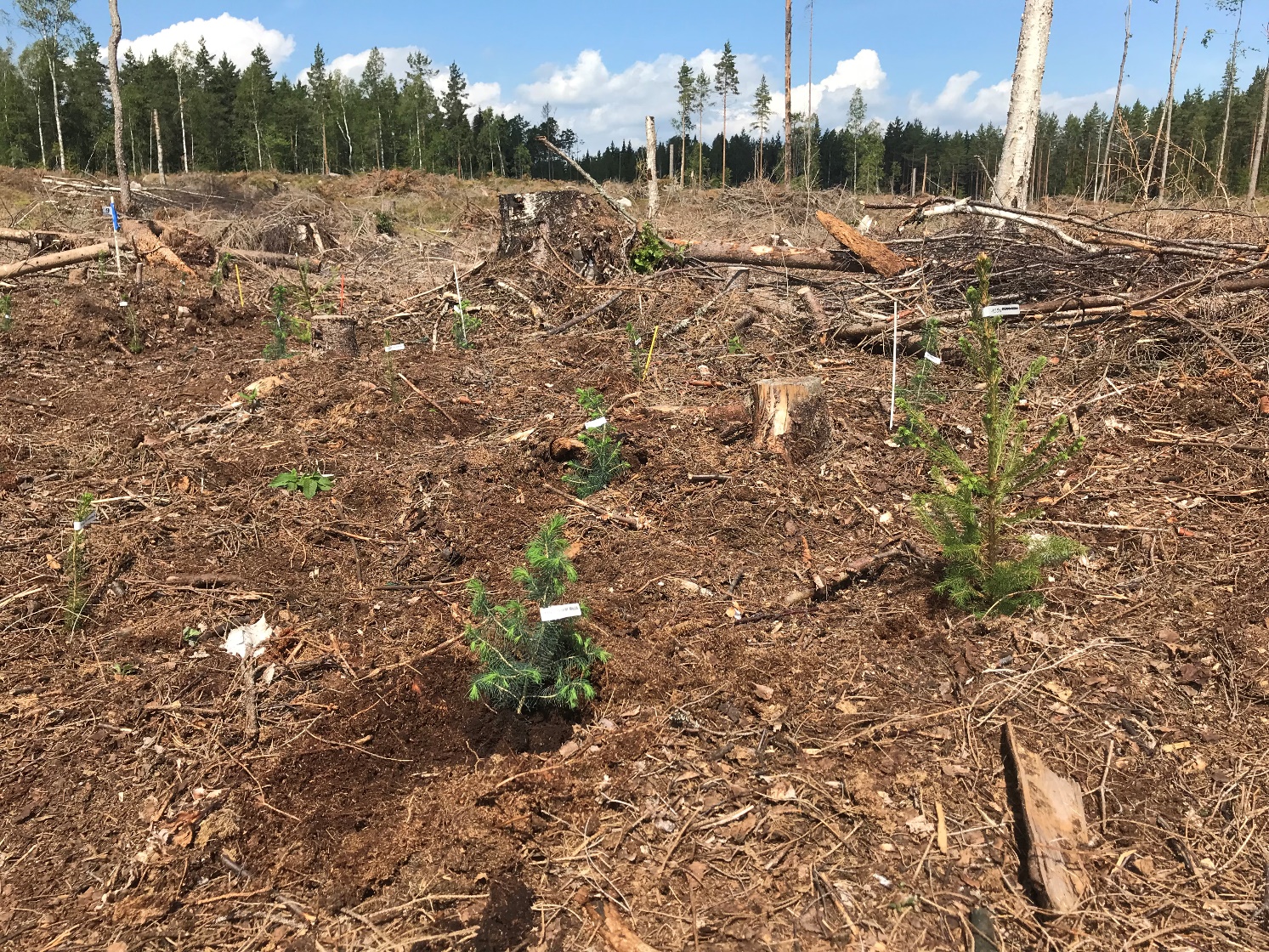


**Figure S4.** Picture of one of the blocks in the field experiment. Norway spruce (*Picea abies*) plants were planted in 9 blocks close to each other in an open clear-cut. The clear-cut was surrounded by mixed-tree forests dominated by Scots pine, as seen in the background. Just south of the experimental area, less than 100 meters from the experimental blocks, there was another regeneration site with young Norway spruce (trees ~10 years old). Small groves were scattered on the clear-cut, but there were no trees close enough to give shade to the experimental blocks. Debris such as cut branches and stems were left on the clear-cut, but large pieces were removed from the experimental blocks. Stumps of different sizes were also found inside in the blocks.


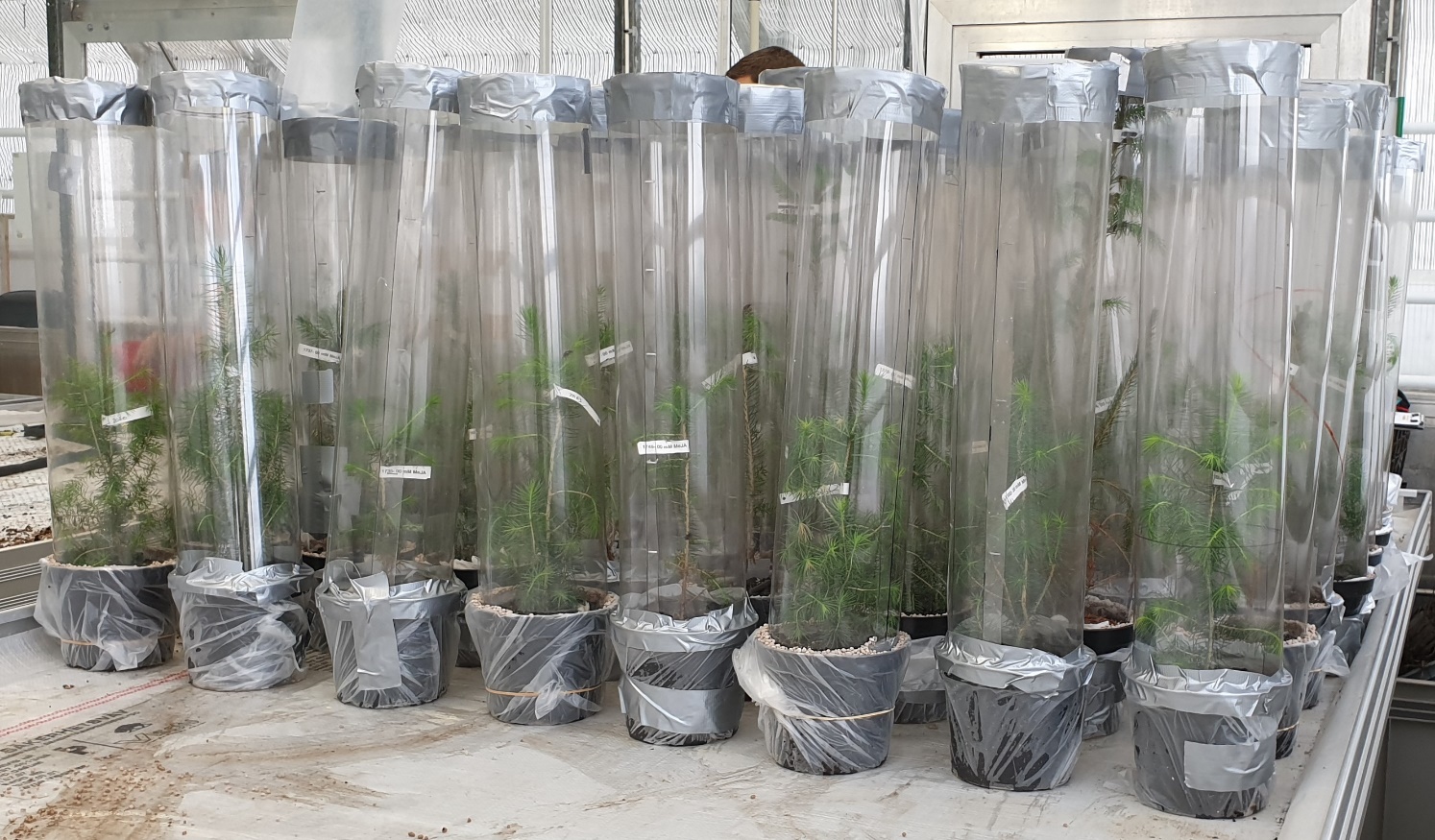


**Figure S5.** Picture of one of the rounds in the no-choice lab experiment. Norway spruce (*Picea abies*) plants and pine weevils (*Hylobius abietis*) were enclosed in plastic cylinders, and placed close to each other on a table. Cylinders and pots were carefully sealed and enclosed by plastic bags to hinder pine weevils from escaping. No artificial lamps were used, so the experiment occurred under natural light conditions coming in through the windows and at room temperature. Plants were not watered during the duration of each round (3-4 days). New plants, pine weevils and plastic bags were used in each round, and water for pine weevils was refilled, but cylinders and pebbles were re-used.


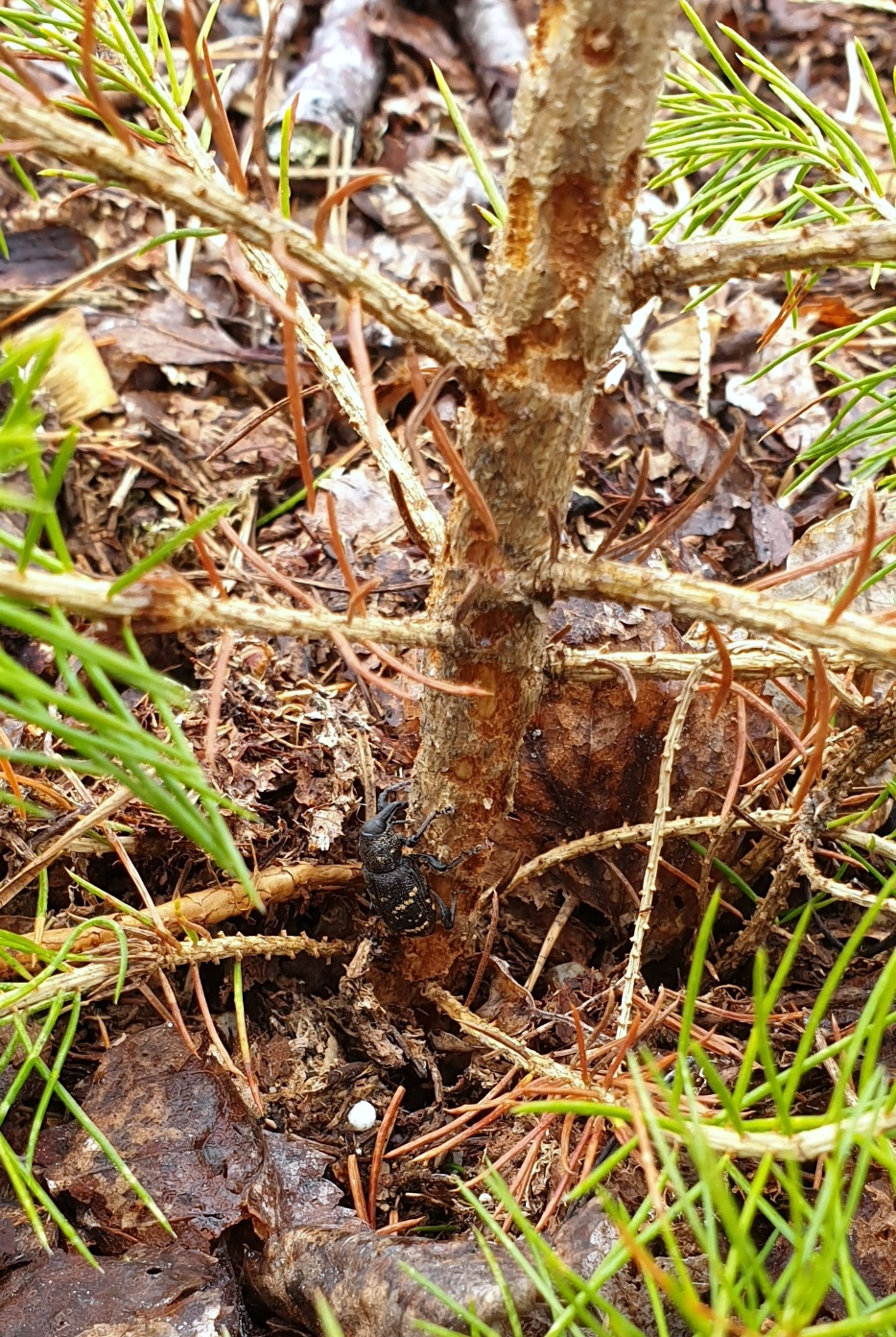


**Figure S6.** Picture of a pine weevil feeding on one of the bare-root plants in the field during year 2.


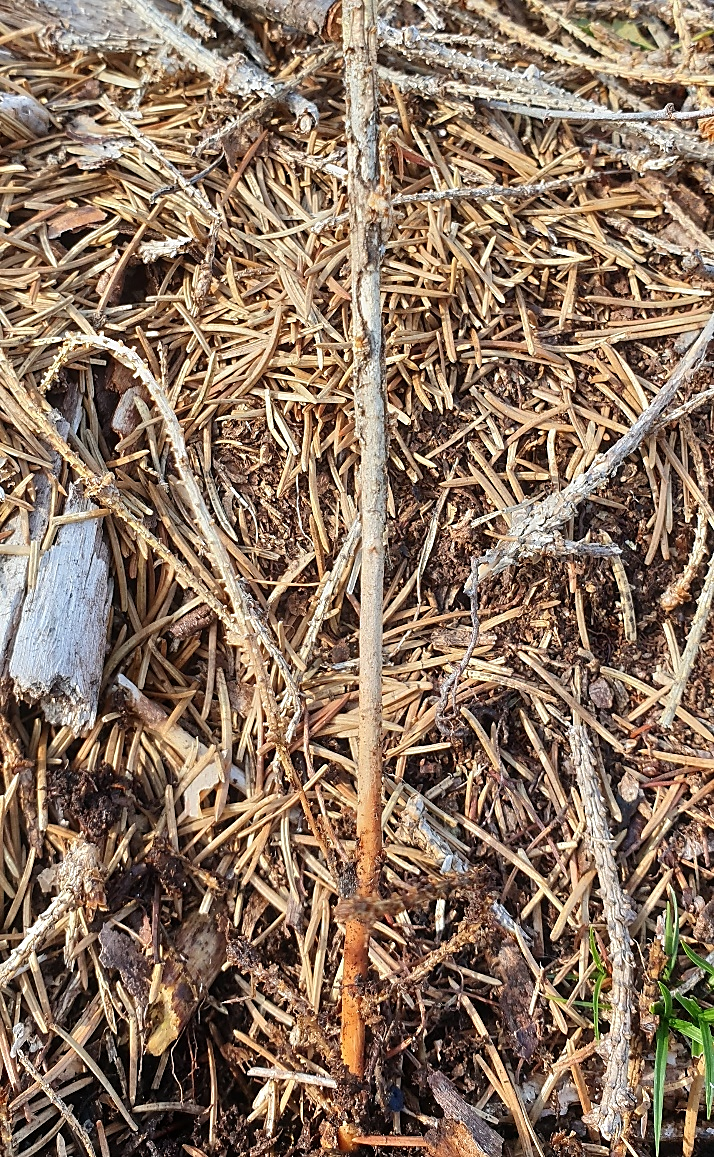


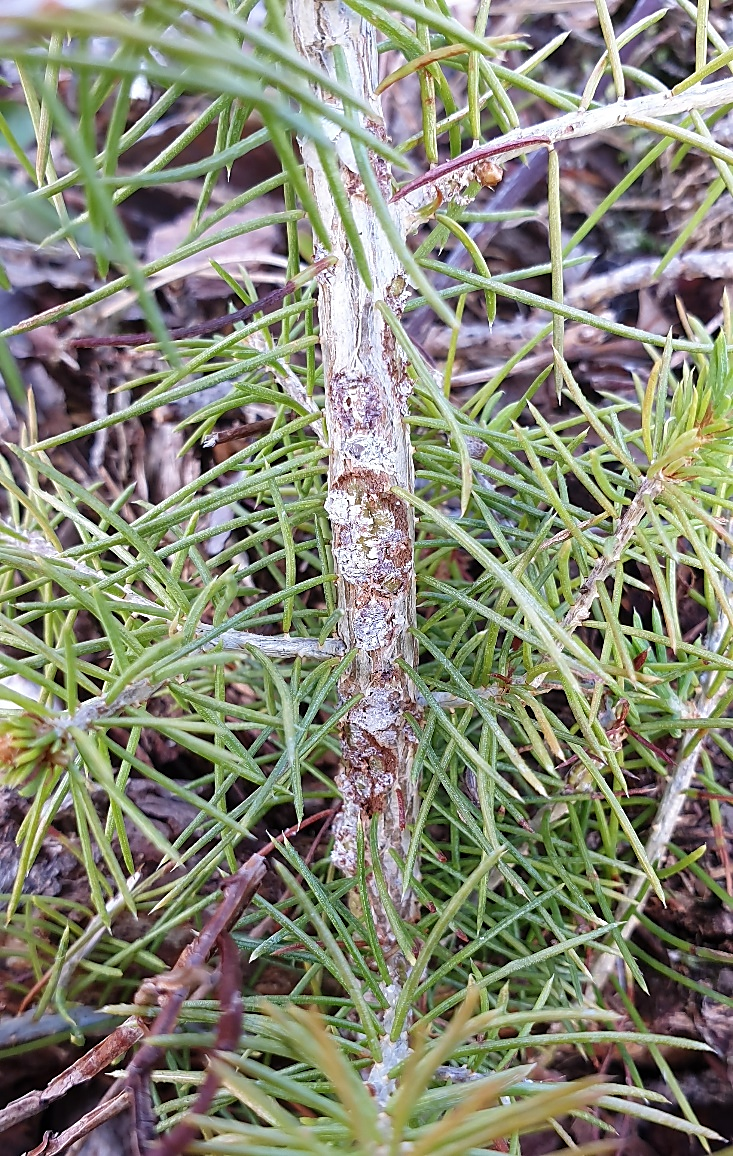


**Figure S7.** Pictures from the field experiment showing pine weevil feeding damage for a non-treated containerized seedling to the left and a MeJA-treated embling to the right. Note that the containerized seedling is completely debarked (no bark left on the lower part of the stem). Pictures were taken in September the first year.

## Supplementary Tables

**Table S1.** Pairwise comparisons between treatment means (z- or t-ratio, p-value) for the proportion of Norway spruce (*Picea abies*) plants attacked and damaged (area debarked, cm^2^) by pine weevils (*Hylobius abietis*), as well as plant mortality (proportion that died) by September 2019, year 1 of the field experiment. Treatments represent the following plant type × MeJA treatment combinations: C = containerized seedlings, B = bare-root seedlings, SE = emblings (produced via somatic embryogenesis), treated (or not) with methyl jasmonate (MeJA: 10 mM). Non-relevant comparisons were excluded, hence, p-values were adjusted for 9 tests instead of 15 (the total) for attack and area debarked. For mortality, p-values were adjusted for 7 tests since MeJA-treated bare-root plants did not experience any mortality the first year. Significant effects (p <0.05) are in bold.

| Field year 1 | Attack | | Area debarked | | Mortality | |
| --- | --- | --- | --- | --- | --- | --- |
|  | z-ratio | p-value | t-ratio | p-value | z-ratio | p-value |
| C vs. SE | -0.889 | 0.9291 | 3.177 | **0.0124** | 5.341 | **<0.0001** |
| C vs. C+MeJA | 0.396 | 0.9981 | 5.829 | **<0.0001** | 5.742 | **<0.0001** |
| C vs. SE+MeJA | 2.469 | 0.0947 | 16.144 | **<0.0001** | 9.147 | **<0.0001** |
| B vs. SE | -2.950 | **0.0248** | 1.601 | 0.5163 | -2.705 | **0.0347** |
| B vs. B+MeJA | -0.467 | 0.9959 | 4.472 | **0.0001** | - | - |
| B vs. SE+MeJA | -1.428 | 0.6390 | 8.416 | **<0.0001** | 2.832 | **0.0244** |
| SE vs. SE+MeJA | 3.388 | **0.0057** | 14.628 | **<0.0001** | 6.966 | **<0.0001** |
| C+MeJA vs. SE+MeJA | 2.122 | 0.2112 | 10.092 | **<0.0001** | 6.508 | **<0.0001** |
| B+MeJA vs. SE+MeJA | -1.055 | 0.8638 | 4.870 | **<0.0001** | - | - |

**Table S2.** Pairwise comparisons between treatment means (z- or t-ratio, p-value) for the proportion of Norway spruce (*Picea abies*) plants attacked and damaged (area debarked, cm^2^) by pine weevils (*Hylobius abietis*) in the lab (July-August 2019). Treatments represent the following plant type × MeJA combinations: C = containerized seedlings, B = bare-root seedlings, SE = emblings (produced via somatic embryogenesis), treated (or not) with methyl jasmonate (MeJA: 10 mM). Non-relevant comparisons were excluded, hence, p-values were adjusted for 9 tests instead of 15 (the total) for area debarked. For attack, p-values were adjusted for 6 tests since non-treated bare-root plants were all attacked in the lab. Significant effects (p <0.05) are in bold.

| Lab experiment | Attack | | Area debarked | |
| --- | --- | --- | --- | --- |
|  | z-ratio | p-value | t-ratio | p-value |
| C vs. SE | -1.336 | 0.5990 | -4.034 | **0.0005** |
| C vs. C+MeJA | -2.037 | 0.1900 | 2.788 | **0.0403** |
| C vs. SE+ MeJA | 1.327 | 0.6052 | 6.508 | **<0.0001** |
| B vs. SE | - | - | -1.660 | 0.4721 |
| B vs. B+MeJA | - | - | -0.266 | 0.9997 |
| B vs. SE+MeJA | - | - | 2.527 | 0.0810 |
| SE vs. SE+MeJA | 2.464 | 0.0693 | 11.786 | **<0.0001** |
| C+MeJA vs. SE+MeJA | 2.949 | **0.0172** | 4.124 | **0.0004** |
| B+MeJA vs. SE+MeJA | -0.517 | 0.9802 | 2.841 | **0.0345** |

**Table S3.** Pairwise comparisons between treatment means (z-ratio, p-value) for the non-cumulative plant mortality (proportion that died) by years 2 and 3 (September 2019 and 2021, respectively), as well as cumulative mortality for years 1-3 (September 2019-2021) in the field. Treatments represent the following plant type × MeJA combinations: C = containerized seedlings, B = bare-root seedlings, SE = emblings (produced via somatic embryogenesis), treated (or not) with methyl jasmonate (MeJA: 10 mM). For cumulative mortality, non-relevant comparisons were excluded, hence, p-values were adjusted for 9 tests instead of 15 (the total). No comparisons were excluded from the non-cumulative analyses. Significant effects (p <0.05) are in bold.

| Mortality field | Year 2  *non-cumulative* | | Year 3  *non-cumulative* | | Years 1-3  *cumulative* | |
| --- | --- | --- | --- | --- | --- | --- |
|  | z-ratio | p-value | z-ratio | p-value | z-ratio | p-value |
| C vs. SE | - | - | - | - | 2.933 | **0.0241** |
| C vs. C+MeJA | - | - | - | - | 2.924 | **0.0249** |
| C vs. SE+MeJA | - | - | - | - | 7.823 | **<0.0001** |
| B vs. SE | -2.517 | 0.0574 | 1.425 | 0.4834 | -1.532 | 0.5513 |
| B vs. B+MeJA | 1.207 | 0.6225 | 1.746 | 0.2996 | 2.788 | **0.0375** |
| B vs. SE+MeJA | 0.768 | 0.8691 | 2.200 | 0.1233 | 2.803 | **0.0358** |
| SE vs. B+MeJA | 3.319 | **0.0050** | -0.370 | 0.9827 | - | - |
| SE vs. SE+MeJA | 6.438 | **<0.0001** | 1.035 | 0.7291 | 9.318 | **<0.0001** |
| C+MeJA vs. SE+MeJA | - | - | - | - | 8.246 | **<0.0001** |
| B+MeJA vs. SE+MeJA | -0.030 | 1.0000 | 1.054 | 0.7175 | 0.745 | 0.9631 |

## Supplementary Text

**Calculations of additive, synergistic or antagonistic effects of SE and MeJA**

To determine the magnitude of the effect on plant resistance (area debarked, first year in the field) when MeJA and SE occur together, we calculated if the effect was additive, synergistic or antagonistic. To do this, we compared observed effects (Obs) with expected effects (Exp) on resistance relative to both untreated containerized and untreated bare-root seedlings (separate calculations) based on Bansal et al. (2013) as follows:

1) Calculation of observed effect sizes of SE and MeJA together on area debarked

$$\text{Obs}\text{ = }\frac{\text{Observed value – }\text{x̄}\text{control}}{\text{x̄}\text{control}}$$

We calculated the observed effect (Obs) on area debarked for emblings that were treated with MeJA (i.e., received SE + MeJA together) relative to untreated containerized seedlings (i.e., not produced via SE, not treated with MeJA). Mean area debarked for non-treated containerized seedlings (x̄_control_) was subtracted from each of the observed area debarked values for each individual MeJA-treated embling, and then divided by x̄_control_. For x̄_control_, we used the estimated marginal means obtained from the model output in the statistical analyses. Absolute values of Obs were used in subsequent steps.

2) Calculation of individual effect sizes (Ind) of SE and MeJA alone on area debarked

$$\text{Ind}\text{ = }\frac{\text{x̄}\text{treatment}\text{ – }\text{x̄}\text{control}}{\text{x̄}\text{control}}$$

We first calculated the individual effect of SE on area debarked for non-treated emblings (i.e., produced via SE only, no MeJA treatment), relative to untreated containerized seedlings (i.e., not produced via SE, not treated with MeJA). Separately, we then calculated the individual effect of MeJA on area debarked for MeJA-treated containerized seedlings (i.e., not produced via SE, received only MeJA treatment). Mean area debarked for non-treated containerized seedlings (x̄_control_) was subtracted from the mean values (x̄_treatment_) of area debarked for non-MeJA treated emblings and MeJA-treated containerized seedlings separately, then divided with the mean area debarked for non-treated containerized seedlings (x̄_control_). We used the estimated marginal means obtained from the model output in the statistical analyses. Absolute values of Ind were used in subsequent steps.

3) Calculation of the expected effect size of SE and MeJA together on area debarked using a multiplicative risk model (to avoid over-inflated response estimates, as outlined by Bansal et al. 2013)

$$\text{Exp}\text{ = }\text{Ind}\text{SE}\text{ + }\text{Ind}\text{MJ}\text{ – (}\text{Ind}\text{SE}\text{ }\text{×}\text{ }\text{Ind}\text{MJ}\text{)}$$

Here, Ind_SE_ is the individual effect size of SE on area debarked that was estimated for non-treated emblings in step 2 above. Likewise, Ind_MJ_ is the individual effect size of MeJA on area debarked that was estimated for MeJA-treated containerized seedlings in step 2 above. The product of the two individual effects was subtracted from the sum of the individual effects.

4) Calculation of the difference between observed and expected effect sizes, with confidence intervals

$$\text{Obs}\text{ – }\text{Exp}$$

The expected effect size calculated in step 3 above was subtracted from each of the observed effect sizes calculated in step 1 for MeJA-treated emblings (i.e., SE + MeJA occurring together). A mean value of these differences was calculated (as well as a ± 95% confidence interval), to determine additive, synergistic or antagonistic effects. If the mean difference between the observed and the expected effect was positive (Obs – Exp > 0), the effect was either additive or synergistic. Further, if the lower limit of the 95% confidence interval was greater than zero, the effect was synergistic, while if it crossed the zero line, it was additive. On the other hand, if the mean difference between the observed and the expected effect was negative (Obs – Exp < 0) and the upper limit of the 95% confidence interval was below zero, the effect was antagonistic. The above four steps were repeated using bare-root seedlings as control.


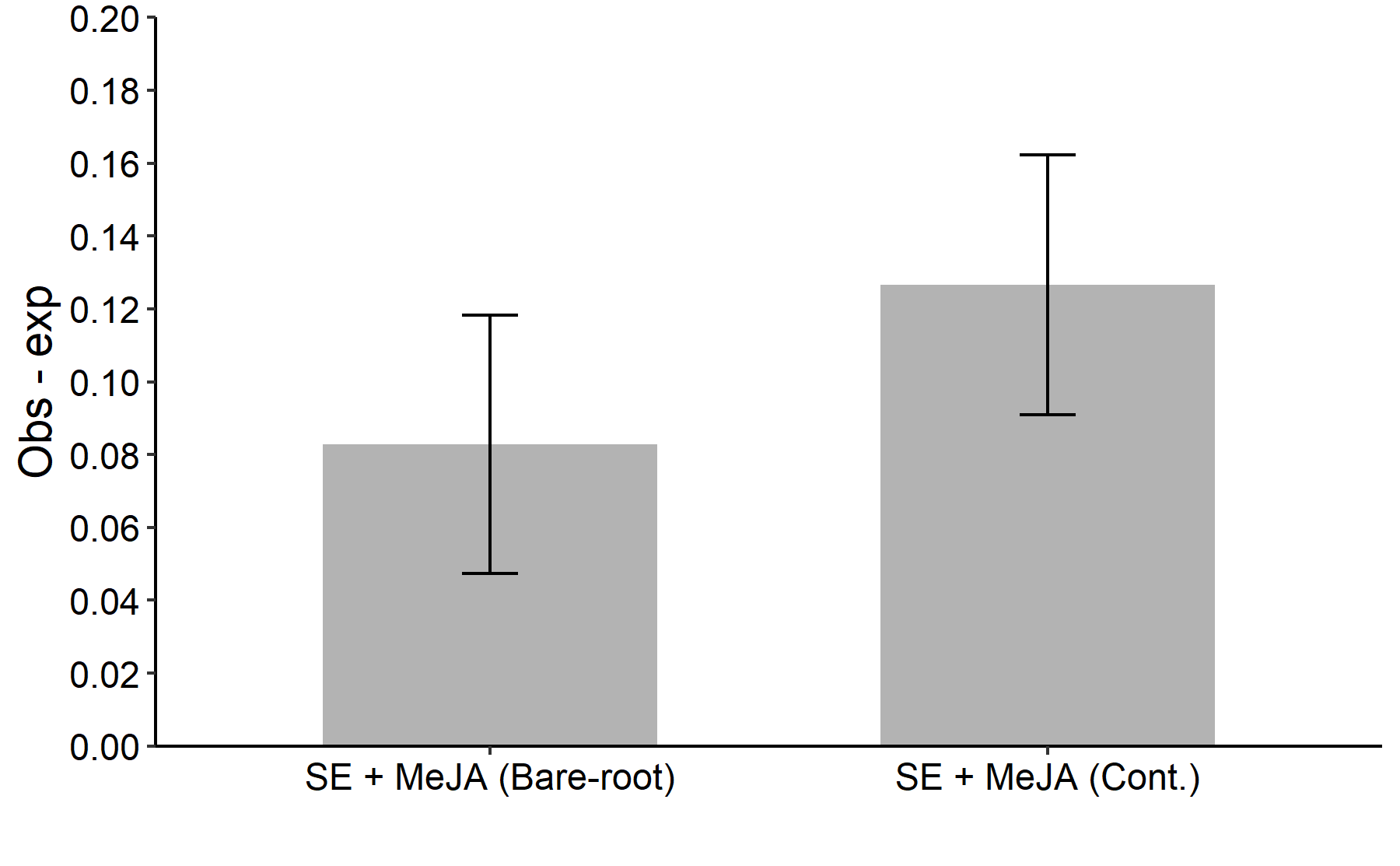


**Figure S8.** Mean differences between observed and expected effects (mean ± 95% CI) of SE and MeJA together on pine weevil (*Hylobius abietis*) damage (area debarked, cm^2^) to Norway spruce (*Picea abies*) seedlings. Bars represent effects calculated relative to the two types of control plants included in the experiment: non-treated bare-root seedlings (left bar) and containerized seedlings (right bar). Given that the means and the 95% confidence limits are greater than zero, the combined effect of SE and MeJA is considered to be greater than the sum of the two independent variables (i.e., effects are synergistic).
